# New Medical Journal

**Published:** 1853-09

**Authors:** 


*New Medical Journal.— The Peninsular Journal of Medicine*, is the title of a new medical periodical, of 48 pages, to be issued monthly at Ann Arbor, Michigan. It is edited by E. Andrews, A. M., M. D., Demonstrator of Anatomy in the University of Michigan. Terms, two dollars per annum.

